# Computational investigation of blood cell transport in retinal microaneurysms

**DOI:** 10.1371/journal.pcbi.1009728

**Published:** 2022-01-05

**Authors:** He Li, Yixiang Deng, Konstantina Sampani, Shengze Cai, Zhen Li, Jennifer K. Sun, George E. Karniadakis

**Affiliations:** 1 School of Engineering, Brown University, Providence, Rhode Island, United States of America; 2 Beetham Eye Institute, Joslin Diabetes Center, Boston, Massachusetts, United States of America; 3 Department of Medicine, Harvard Medical School, Boston, Massachusetts, United States of America; 4 Division of Applied Mathematics, Brown University, Providence, Rhode Island, United States of America; 5 Department of Mechanical Engineering, Clemson University, Clemson, South Carolina, United States of America; 6 Department of Ophthalmology, Harvard Medical School, Boston, Massachusetts, United States of America; Stanford University, UNITED STATES

## Abstract

Microaneurysms (MAs) are one of the earliest clinically visible signs of diabetic retinopathy (DR). MA leakage or rupture may precipitate local pathology in the surrounding neural retina that impacts visual function. Thrombosis in MAs may affect their turnover time, an indicator associated with visual and anatomic outcomes in the diabetic eyes. In this work, we perform computational modeling of blood flow in microchannels containing various MAs to investigate the pathologies of MAs in DR. The particle-based model employed in this study can explicitly represent red blood cells (RBCs) and platelets as well as their interaction in the blood flow, a process that is very difficult to observe *in vivo*. Our simulations illustrate that while the main blood flow from the parent vessels can perfuse the entire lumen of MAs with small body-to-neck ratio (BNR), it can only perfuse part of the lumen in MAs with large BNR, particularly at a low hematocrit level, leading to possible hypoxic conditions inside MAs. We also quantify the impacts of the size of MAs, blood flow velocity, hematocrit and RBC stiffness and adhesion on the likelihood of platelets entering MAs as well as their residence time inside, two factors that are thought to be associated with thrombus formation in MAs. Our results show that enlarged MA size, increased blood velocity and hematocrit in the parent vessel of MAs as well as the RBC-RBC adhesion promote the migration of platelets into MAs and also prolong their residence time, thereby increasing the propensity of thrombosis within MAs. Overall, our work suggests that computational simulations using particle-based models can help to understand the microvascular pathology pertaining to MAs in DR and provide insights to stimulate and steer new experimental and computational studies in this area.

## Author summary

Microaneurysms (MAs) are one of the earliest clinically visible signs of diabetic retinopathy (DR). MA leakage or rupture may precipitate local pathology in the surrounding neural retina that impacts visual function. In this work, we perform computational modeling of blood flow in microchannels containing various MAs to investigate the pathologies of MAs in DR. The particle-based model employed in this study can explicitly represent red blood cells (RBCs) and platelets as well as their interaction in the blood flow, a process that is very difficult to observe *in vivo*. Our simulations illustrate that while the main blood flow from the parent vessels can perfuse the entire lumen of small MAs, it can only perfuse part of the lumen in large MAs. Our results also show that enlarged MA size, increased blood velocity and hematocrit in the parent vessel of MAs as well as the RBC-RBC adhesion promote the migration of platelets into MAs, thereby increasing the propensity of thrombosis within MAs. Overall, our work suggests that computational simulations using particle-based models can help to understand the microvascular pathology pertaining to MAs in DR and provide insights to stimulate and steer new experimental and computational studies in this area.

## Introduction

**Fig 1:**
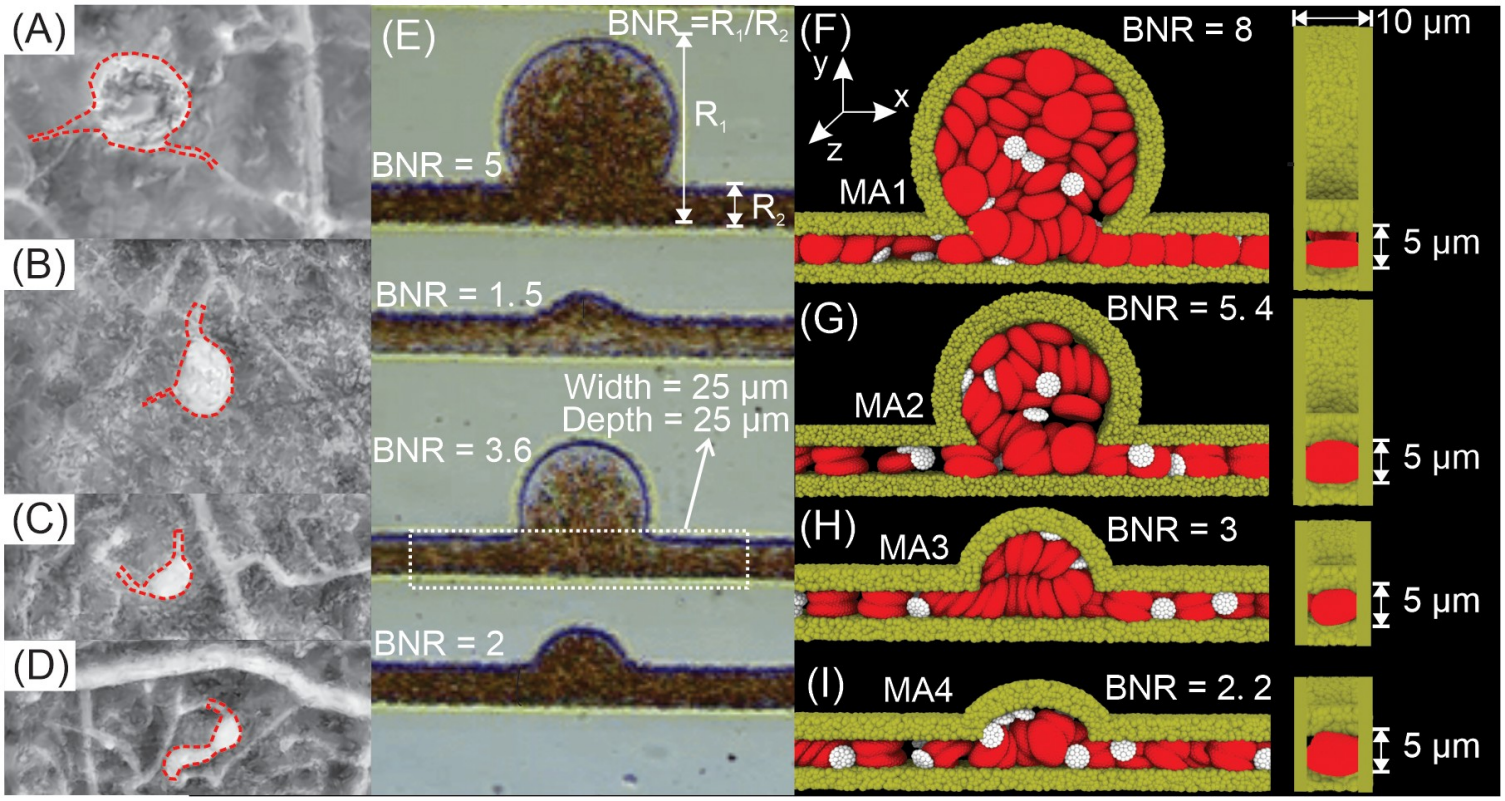
(A-D) Left, four MAs with varying sizes are imaged using AOSLO multiply scattered light imaging modality. Red dotted curves signify the boundary of the MAs and their parent capillaries. (E) Microfluidic channels were fabricated in [54] in order to study the blood flow in MAs with different sizes. The size of the MA is characterized by the body-to-neck ratio (BNR), which is defined as the largest dimension of the MA body (*R*_1_) divided by the diameter of the parent vessel (*R*_2_). Figure is adopted from [54] with permission. (F)-(I) Four MA channels (left: front view, right: side view) with different BNRs (8, 5.4, 3 and 2.2) are devised to mimic blood vessels with MAs. The simulated blood consists of RBCs (red), platelets (white) and solvent particles (not shown for clarity). RBCs are initially placed inside the microchannel whereas the platelets are flowing in from the right inlet of the microchannels when simulations start (flow is from right to left). The hematocrit of 10%, 20% and 40% are examined in the simulations.

Microaneurysm (MAs) are one of the earliest clinical signs detected by the routine fundus examination for diabetic retinopathy (DR) [1], the most common microvascular complication of diabetes. Despite advances in systemic control and intraocular treatments, DR remains a leading cause of visual impairment and blindness globally [2]. MA counts and severity are widely used as an indicator of DR severity level and of DR worsening while MA turnover rate has been associated with risk of future progression of DR [3–5]. Leakage or rupture of MAs result in retinal edema or hemorrhage, which can directly affect retinal function. Recent advances in adaptive optics scanning laser ophthalmoscopy (AOSLO) provide high-resolution images of MAs *in vivo* (see examples in Fig 1A–1D) that enables classification of MA’s morphologies into various categories, including focal bulging, saccular, fusiform, mixed saccular/fusiform, pedunculated and irregular-shaped MAs [6]. Bernabeu et al. [7] used AOSLO to differentiate the thrombus-filled region from the perfused region inside individual MAs and found that thrombi are more frequently observed in saccular-shaped compared to fusiform-shaped MAs. This finding can be used to explain the clinical evidence that some MAs disappear in the retinas of diabetic patients over time, which could be attributed to the complete thrombosis of the dilated lumen [5, 8]. However, the underlying mechanism that initiates thrombosis in MAs is not clear. Data reported from clinical and computational studies [9–11] imply that thrombosis in MAs may be associated with their risk of leakage. Therefore, a detailed investigation of the mechanism of thrombus formation in MAs and their propensity to present in certain group of MAs could improve our understanding of the pathophysiology of MAs in DR.

Platelet transport in circulation plays an essential role in primary hemostasis and platelet plug formation. Under physiological conditions, red blood cells (RBCs) in blood flow tend to form a cell-rich region in the core of the vessel and expel platelets toward a cell-free layer near the vessel wall [12]. The margination of platelets contributes to their quick activation and accumulation at the injure sites on the vessel wall, an initial process of blood clotting to stop bleeding. Extensive studies have been performed to investigate platelet margination at various vessel sizes, blood hematocrit and flow shear rates through *in vivo* [13–15], *in vitro* [16–19] and *in silico* methods [20–23]. Under pathological conditions, alterations in blood vessels could affect the transport of blood cells and precipitate undesired thrombus formation. A number of *in vivo* [24, 25] and *in vitro* experiments [25–28] have demonstrated that platelets flowing in the vessels or channels containing stenoses are prone to aggregate at the poststenotic sites. These experimental observations were elaborated by a prevailing hypothesis [28] that after passing stenosis apex, RBCs tend to migrate toward the wall and thus collide with platelets and force them move close the wall, causing platelet post-stenosis deposition. This hypothesis was confirmed by subsequent computational modeling of RBCs and platelets flowing in channels with constrictions [29–31]. Yazdani and Karniadakis [29] further demonstrated that higher levels of constriction and the ensuing increased wall shear rates could enhance post-stenosis platelet margination.

In contrast to the significant progress made in elucidating the dynamics of blood cells in normal vessels and vessels with stenoses, transport of platelets in vessels containing aneurysms has not been investigated in detail. Thrombi have been detected in various types of aneurysms both at the macro-scale, such as cerebral aneurysms [32], coronary artery aneurysms [33], abdominal aortic aneurysms [34] and at the micro-scale, like MAs [7], but prior experimental studies [35–38] in this context have been mainly focused on the influence of intraluminal thrombus on the stability of the aneurysm wall in macroscale aneurysms with little effort made to understand how platelets transport and deposit in these aneurysms. On the other hand, computational models have been used to simulate the transport and distribution of RBCs and platelets in channels with aneurysmal geometries at macroscale [39–46]. These existing models either treat platelets as a mean field concentration [39, 40] or model them as tracer particles in a homogeneous fluid [41–43] without explicitly simulating RBCs. The effect of RBCs on platelet motion is simplified by enhancing platelet diffusive motion toward the wall [44–46]. Computational models with these simplifications can produce results in a good agreement with experimental data in simple channel flows [43, 47–49], but they may not perform well in case of complex flow geometries [50–52]. In particular, no computational studies have been conducted to investigate the transport of RBCs and platelets in microchannels or vessels containing MAs.

Although *in vivo* AOSLO imaging, as shown in Fig 1A–1D, helps ophthalmologists to evaluate individual MA’s morphologies [6], detect the thrombi inside MA [7] and assess the blood velocity in the parent capillaries [53], it does not provide the resolution to monitor the motion of individual blood cells flowing within MAs. A recent *in vitro* study has attempted to investigate the dynamics of RBCs and platelets transporting in microchannels containing MAs [54] (see Fig 1E). However, in this work, the size of the parent channels of the MAs was ∼25*μm*, which is several-fold larger than the size of retinal capillaries which usually range from 5 to 15*μm* in diameter [55]. This discrepancy may result in different hemodynamics inside these MAs compared to those found under normal physiological conditions. In the current work, we simulate the traveling of RBCs and platelets in the microchannels containing various aneurysmal geometries. The sizes of MAs and their parent vessels are designed to be comparable to those observed *in vivo* (see Fig 1A–1D). As displayed in Fig 1F–1I, the microchannels are devised to mimic saccular-shaped MAs, the most prevalent shape of MAs observed in the retinal microvasculature of DR patients [6, 10]. We employ a particle-based model to simulate blood flow with explicit representation of RBCs and platelets in order to investigate the role of RBC-platelet interaction in the blood cell transport within MAs. We also quantify the impacts of the size of MAs, blood flow velocity, the hematocrit levels and the biomechanics of RBCs on the dynamics of platelets inside the MA to explore how these factors contribute to the high propensity of thrombosis in saccular-shaped MAs.

## Models and methods

### Particle-based models of blood flow

#### Blood plasma

The blood plasma in the simulation is modeled using dissipative particle dynamics (DPD) method following our previous paper [56]. Detailed information of the DPD method can be found in S1 Text and model parameters are listed in Table A in S1 Text.

#### RBC and platelet models

In the last two decades, numerous multiscale RBC models have been developed to investigate the biological processes associated with RBCs from the protein-level to cellular level, see recent reviews [57–60]. Although the protein-level RBC models, such as [61–64], can be used to assess the altered mechanical properties and morphologies of RBCs induced by either protein defects in blood disorders or virus invasion [65–70], but it is still computationally prohibitive to simulate blood cell suspension or blood flow in the microvessels. Thus, in this work, we employ an efficient cellular level model developed using DPD method to represent RBCs [71, 72] and use its extension for platelets [29]. In this model, the cell membrane is defined as a set of *N*_*v*_ DPD particles with Cartesian coordinates *X*_*i*_, *i* ∈ 1, …, *N*_*v*_ in a two-dimensional triangulated network. The free energy of the system is given by

$$\begin{array}{c} V_{t}=V_{s}+V_{b}+V_{a}+V_{v} \end{array}$$ (1)

where *V*_*s*_ is the stored elastic energy associated with the worm-like-chain (WLC) bonds between DPD particles, *V*_*b*_ is the bending energy of the cell membrane, and *V*_*a*_ and *V*_*v*_ are the energies designed for cell surface area and volume constraints, respectively. The formulation of these energies and the parameters in the model can be found in the S1 Text. In this study, we model a normal RBC with *N*_*v*_ = 500, shear modulus *μ*_0_ = 4.73 *μ*N/m and bending rigidity *k*_0_ = 2.4×10^−19^ J. The cell surface area is selected to be $A_{0}^{\text{tot}}=132.9\;{\mu m}^{2}$, and cell volume $V_{0}^{\text{tot}}=92.5\;{\mu m}^{2}$, which give S/V = 1.44. All parameters used in our normal RBCs model are calibrated based on existing experimental data from single RBC mechanics to blood flow dynamics [23, 73–76].

In the case of platelets which are nearly rigid in their passive form, we choose shear modulus and bending rigidity sufficiently large (100 times larger than the normal RBCs) to ensure its rigid behavior. The number of vertices in the platelet’s membrane network is *N*_*v*_ = 48 and the aspect ratio of the cell is *AR* = 0.38. Based on our previous analysis of patient-specific data [23, 76], a normal platelet has cell volume $V_{0}^{\text{tot}}=6\;{\mu m}^{2}$. All the parameters in the DPD RBC and platelets models are listed in Tables B and C in S1 Text.

#### RBC-RBC adhesion model

Prior studies have suggested that enhanced RBC-RBC adhesion in diabetic blood could precipitate RBC aggregation and rouleau formation in the microcirculation [77]. In our simulation, we also consider the effect of RBC-RBC adhesion on the platelet transport within MAs. Following our previous computational study of the RBC adhesion and rouleau formation in diabetic blood [78], we employed a Morse potential to model RBC-RBC interactions and it is expressed as

$$\begin{array}{c} V_{M}\left(r\right)=D_{e}\left[e^{2\beta (r_{0}-r)}-2e^{\beta (r_{0}-r)}\right]\;, \end{array}$$ (2)

where *r* is the separation distance, *r*_0_ is the zero force distance, *D*_*e*_ is the well depth of the potential, and *β* characterizes the interaction range. The value of the parameters in the Morse potential can be found in Table D in S1 Text. In the original work of [78], the number of adhesive particles on the RBCs is tuned to account for the fibrinogen concentration-dependent intercellular adhesion between RBCs. In the current work, we assume all the particles on the RBCs are adhesive to represent a case with high concentration of fibrinogen in the plasma and emphasize the impact of RBC adhesion on the transport of platelets in MAs.

## Results

To address the diversity in size for clinically observed MAs (see Fig 1A–1D), we construct different *in silico* microfluidic channels shown in Fig 1F–1I. Following the work of Ezra et.al [9], we use the non-dimensional parameter, body-to-neck ratio (BNR), which is defined as the ratio between the diameter of the MA (*R*_1_) and the width of its parent vessel (*R*_2_) (Fig 1E), to quantify the size of MAs. Specifically, we simulate four MAs with BNRs ranging from 2.2 to 8. As shown in Fig 1F–1I, the width of the parent vessels is *R*_2_ = 5 *μm* whereas the depth of the vessels is 10 *μm*, which are comparable with the size of capillaries. We impose periodic boundary conditions along *x* direction and implement arbitrary boundary method [79] on the channel wall to impose the no-slip boundary conditions. RBCs are filled into the microchannel with hematocrit levels of 40%, 20% and 10%, respectively. Platelets are flowing into the channel from the inlet once the simulation starts. To drive the blood flow, we impose a pressure gradient between the inlet and outlet and this pressure gradient is tuned to vary the inlet blood flow velocities from 0.25 mm/s to 2.0 mm/s. Each simulation runs 50,000,000 timesteps (corresponding to 1.3 seconds in the physical time). We quantify two metrics that are associated with thrombus formation in MAs, namely the probability of platelets entering an MA per passage of the microchannel as well as their residence time inside the MA.

### Effect of the MA size on platelet transport

**Fig 2:**
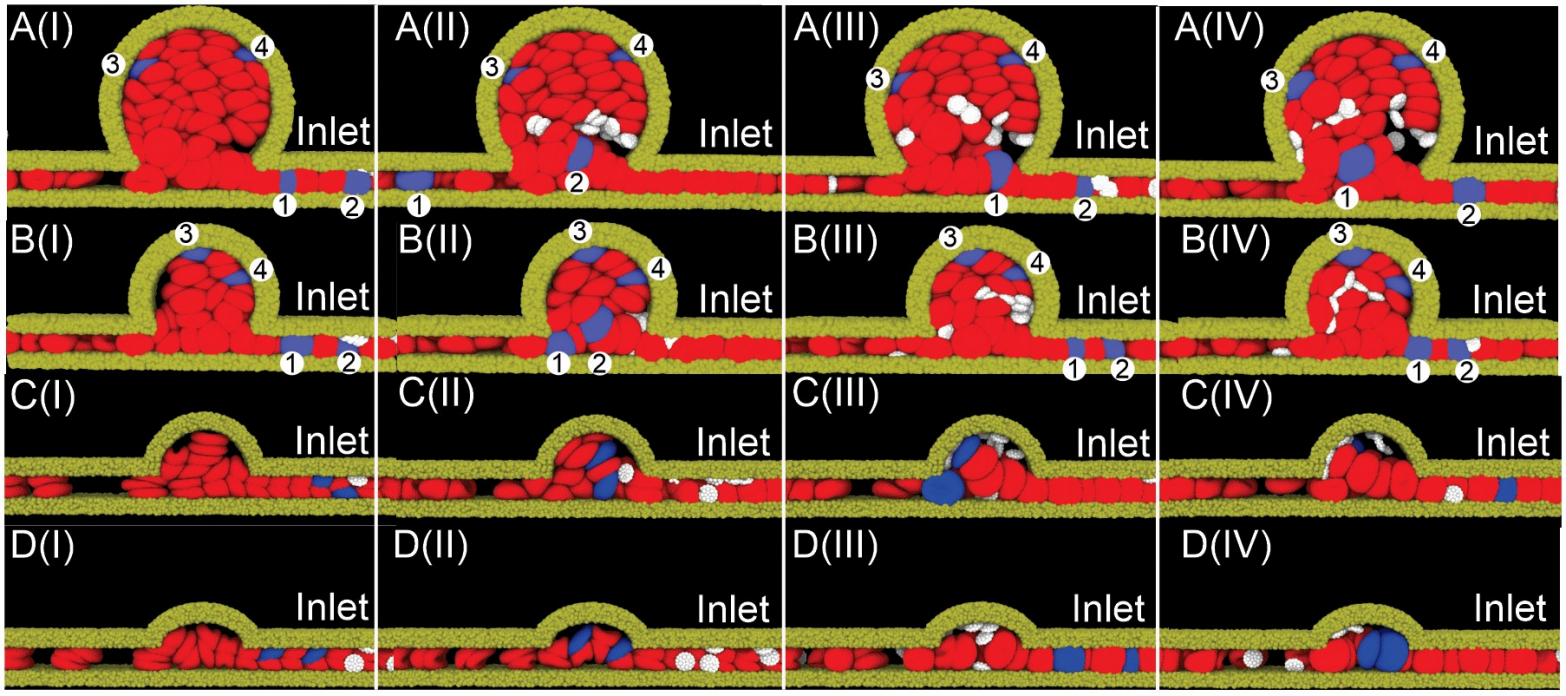
Sequential snapshots of RBCs (red) and platelets (white) traveling in the four MA microchannels with BNR = (A) 8, (B) 5.4, (C) 3, (D) 2.2, during the simulation time of 1.3 seconds. The timestamp increases sequentially from snapshot (I) to (IV). In (A) and (B), four RBCs are highlighted in blue to track their motion during the simulation. No.1 and No.2 RBCs are initially located in the parent channels whereas No.3 and No.4 RBCs are initially located inside MAs. In (C) and (D), two RBCs are highlighted in blue to track their motion during the simulation. The blood flow velocity at the inlet is 1.5 mm/s for all the four MA channels. Solvent particles are omitted for clarity.

First, we examine the effect of the size of MAs on RBC and platelet transport inside the MAs without considering the impact of the RBC adhesion and aggregation. Here, we select the inlet velocity of 1.5 mm/s, which falls within the physiological range of blood flow velocities measured in parafoveal capillaries by De Castro et al [53]. Fig 2A(I)–2A(IV) illustrate four sequential snapshots of RBCs and platelets traveling through the microchannel containing an MA with BNR = 8. We observe that some of the platelets migrate into MAs whereas the two highlighted RBCs initially located in the parent vessel (No.1 and No.2 in blue) travel in and subsequently flow out of MAs without penetrating into the MA lumen. On the other hand, we note that the displacements of the two highlighted RBCs initially placed inside the MA (No.3 and No.4 in blue) are very small, implying that the flow deep inside the lumen of the MA is nearly stagnant. Similar to the observations made from the MA with BNR = 8, Fig 2B(I)–2B(IV) show that the motion of two RBCs initially located inside the MA with BNR = 5.4 (No.3 and No.4 highlighted in blue) is very slow. The two highlighted RBCs originating from the parent channel do not enter the lumen of MA (No.1 and No.2 in blue) whereas the platelets are repelled into the MA lumen at the intersection between the MA and its parent vessel. In case of two MAs with smaller BNRs (BNR = 3 and 2.2), both two highlighted RBCs and platelets can flow into and exist MA lumen repeatedly during the simulation time of 1.3 seconds (see Fig 2C and 2D(I)–2D(IV)).

**Fig 3:**
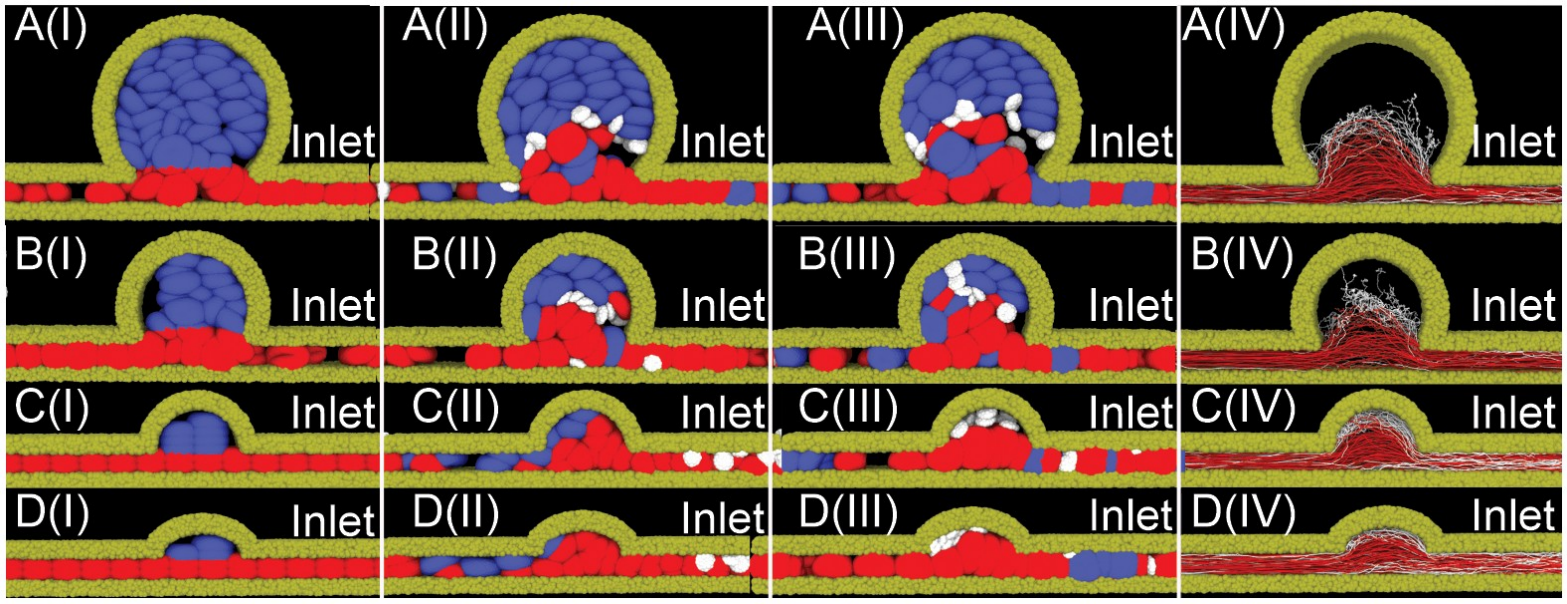
Sequential snapshots of RBCs (red) and platelets (white) traveling in the four MA microchannels with BNR = (A) 8, (B) 5.4, (C) 3, (D) 2.2, during the simulation time of 1.3 seconds. The hematocrit level is 40% in the microchannel. The timestamp increases sequentially from snapshot (I) to (III). No platelets are initially placed in MAs. Solvent particles are omitted for clarity. The corresponding trajectories of RBCs (red curves) and platelets (white curves) are plotted in (IV). Our results illustrate that the main blood flow from the parent channel of the MAs can perfuse the entire lumen of two MAs with smaller BNR shown in (C) and (D), but cannot do so for two MAs with larger BNR shown in (A) and (B). The inlet (right end of the microchannel) blood flow velocity is 1.5 mm/s for all the four MAs.

The bulk motion of RBCs and platelets in these four examined MA channels can be elucidated in Fig 3 where the RBCs initially located within MAs are highlighted in blue while RBCs initially located in the parent channel are highlighted in red. Fig 3A(I)–3A(III) and 3B(I)–3B(III) show that in case of BNR = 8 and 5.4, both RBCs (red) and the platelets flow into the MA lumen from the parent vessels due to the expansion of the channel. Most of RBCs originating from the parent vessels exit the MAs following the main flow. As a result, the MA lumen is mostly occupied by the RBCs initially located inside the MAs (blue). In contrast, many platelets deviate from the main flow and move further into the MA lumen due to their collision with RBCs. This distinct motion of platelets from RBCs within MAs demonstrates the essential role of RBC-platelet interaction on the transport of platelet into the lumen of MAs. The corresponding trajectories of RBCs in Fig 3A(IV) and 3B(IV) illustrate that the main blood flow from the parent channel of the MAs cannot perfuse the entire lumen for MAs with BNR = 8 and 5.4. In case of MAs with smaller lumen (BNR = 3 and 2.2), Fig 3C(I)–3C(III) and 3D(I)–3D(III) illustrate that RBCs initially located inside the MAs (blue) can be quickly transported out of the lumen, indicating that both MA lumen can be fully perfused by the blood from the parent vessels. The trajectory plots in Fig 3C(IV) and 3D(IV) further show that although both RBCs and platelets can enter MA lumen, platelets are moving closer to the vessel wall, facilitating the potential platelet adhesion and aggregation inside the MA.

**Fig 4:**
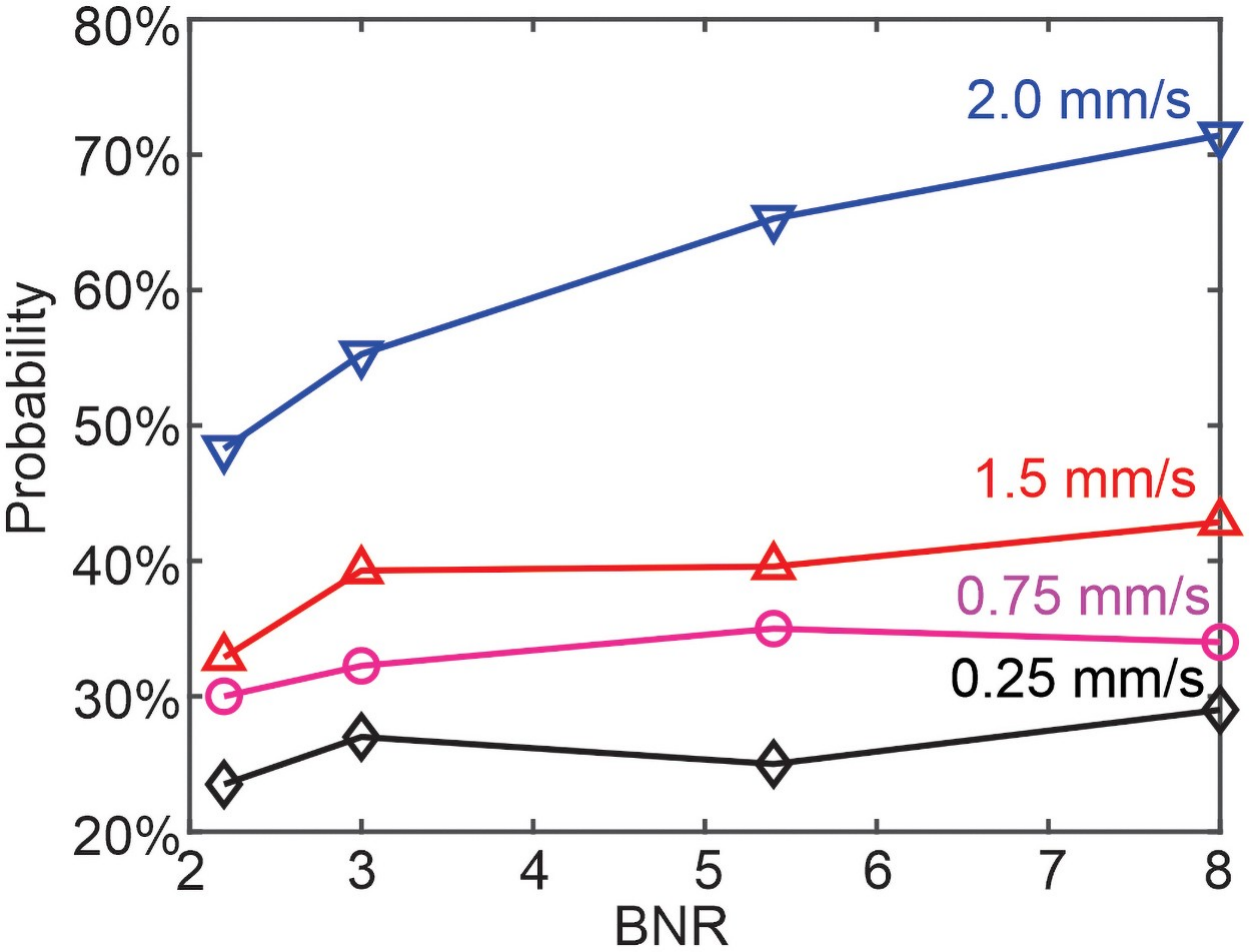
Probability of platelets entering the four examined MAs (MA1, BNR = 8; MA2, BNR = 5.4; MA3, BNR = 3; MA4, BNR = 2.2) under four different inlet velocities. The probability is represented by the ratio between the number of times that the platelets travelled into the MA and the total number of platelet passage through the MA channel. The results suggest that higher inlet velocity leads to larger probability of platelets entering MAs for the four examined MAs.

**Fig 5:**
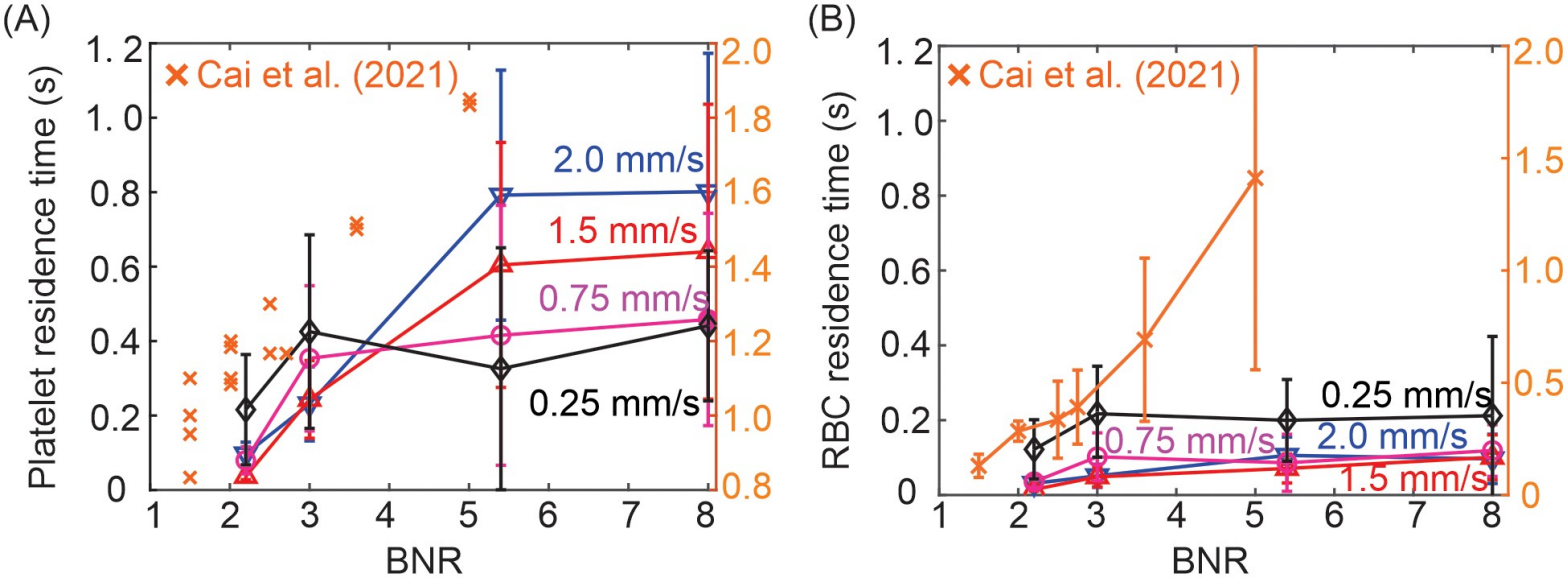
Residence time of (A) platelets and (B) RBCs in four MA microchannels (MA1, BNR = 8; MA2, BNR = 5.4; MA3, BNR = 3; MA4, BNR = 2.2) under different inlet velocities during the simulation time of 1.3 seconds. RBCs that are initially placed in MAs are excluded from the measurements. The residence time is recorded as the time elapse between the moment when RBCs/platelets enter the MAs and the moment when they exist the MAs. The error bars are computed based on measurements of 20 platelets and 26 RBCs from simulations. The orange cross symbols represent the measurements from the microfluidic experiments performed in Cai et al. [54] and their values are represented by y-axis on the right. The scatter points in (A) represent the measurements of the residence time of 15 platelets tracked in six microchannels with varying BNRs (BNR = 1.5, 2, 2.5, 2.75, 3.6 and 5). The error bars in (B) are computed based on measurements of 20 RBCs randomly selected in each of six microchannels examined in [54].

To quantify the transport of the RBCs and platelets in the MA channels, we compute the probability of platelets entering different MAs as well as their residence time within the MAs. Fig 4 shows that the probability of platelets entering the lumen of MAs gradually increases as BNR becomes larger (see red curve that signifies the results measured at inlet velocity of 1.5 mm/s). Furthermore, the red curve in Fig 5A shows that platelets traveling into MAs with a larger BNR have a longer residence time than those entering MAs with smaller BNR. Similar results are also observed for RBCs as shown in Fig 5B (red curve), although the residence time of RBCs in MAs is much shorter than those of platelets. A similar trend of variation of residence time of RBCs and platelets with respect to BNR was also observed from the microfluidics experiments in [54] (see orange cross symbols in Fig 5A and 5B), but the residence times measured from the experiments are greater than the simulation results. The increased residence times of RBCs and platelets in the microfluidics experiments are likely due to the greater size of MA bodies (50–125 *μm* against 11–40 *μm* in our simulations) as well as larger width of MAs’ neck (∼75 *μm* against ∼15 *μm* in our simulations) that introduces an increased amount of blood flow into the MAs from their parent vessels and thus allows full perfusion of MA lumen for all the examined MAs in [54]. However, both the simulation results and experimental measurements suggest that the size of MAs influences the transport of platelets and RBCs into the MAs as well as their residence time, and thus could be associated with the *in vivo* thrombus formation within MAs [6, 7].

### Effect of hemodynamics on platelet transport

In this section, we investigate the role of blood flow velocity in transporting platelets and RBCs in the channels containing MAs without considering the impact of the RBC adhesion and aggregation. The inlet blood flow velocity employed in the last section was adopted from previous measurements of parafoveal capillaries [53] and this velocity can vary among individual patients with diabetes. In addition, retinal hemodynamics progressively change overtime due to capillary dropout [80, 81] and vasodilation [82, 83]. Both increased and decreased retinal blood flows in eyes with DR have been reported from multiple prior studies using different measurement techniques [84]. The current consensus is that total retinal blood flow decreases initially in very early DR and then normalizes and subsequently increases due to vascular shunting in advanced DR. To consider the impact of the hemodynamics, we vary the inlet blood velocity from 1.5 *mm*/*s* to 0.25 *mm*/*s*, 0.75 *mm*/*s*, 2.0 *mm*/*s*, respectively and explore how these varied inlet blood velocities affect the likelihood of platelets entering the four examined MAs as well as their residence time inside.

Fig 4 shows that the increased inlet blood velocity boosts the likelihood of platelets entering the lumen of MAs, which is likely driven by the enhanced collision between the platelets and RBCs at higher blood velocities and thus forces more platelets migrate into the MA lumen, a mechanism also causing the enhanced platelet margination in high-shear flow [21, 85, 86]. Fig 5A shows that once the platelets enter the MAs with larger BNR, i.e., the MAs with BNR = 8 and 5.4, the residence time of the platelets increases with elevated blood flow velocities. However, in case of MAs with smaller BNR, i.e., the MAs with BNR = 3 and 2.2, the residence time of the platelets decreases with increased blood flow velocities. This discrepancy could result from the fact that the amplified collision between platelets and the RBCs due to increased blood flow velocity can propel platelets into the non-perfused region in case of MAs with larger BNR, thereby prolonging their residence time. As for the MAs with smaller BNR, however, Fig 3C and 3D illustrate that the main flow from the parent channel can perfuse the whole MA lumen so a lower blood velocity would allow a longer residence time of platelets inside the MAs. Fig 5B shows that the residence times of RBCs originating from parent vessels are shorter than those of the platelets, particularly for larger MAs (BNR = 8 or BNR = 5.4) as the RBCs barely deviate from the main flow and reside in the lumen of MA.

### Effect of RBC biomechanics on platelet transport

Altered biomechanics of RBCs in diabetic blood leads to enhanced RBC aggregation [77, 87] and abnormal hemorheology [88, 89], contributing to the prothrombotic states experienced by many diabetic patients. In particular, multiple independent studies [90–93] demonstrated that diabetic RBCs are less deformable than their healthy counterparts by showing that the shear modulus of diabetic RBCs was 2∼5 times larger than that of normal RBCs. In addition, the RBC-RBC adhesion is enhanced in diabetic blood, leading to pronounced RBC aggregation and rouleau formation in microcirculation [77, 87]. In this section, we consider the RBC-RBC adhesion and the increased RBC stiffness as reported in the diabetic blood and examine their effects on the platelet transport in MA channels. To account for the RBC-RBC adhesion, we follow our previous computational study of diabetic RBCs [78], where we reproduced the dynamics of rouleau formation and disaggregation observed from *in vitro* experiments. Moreover, we assume an extreme value of stiffness for the diabetic RBCs by selecting the shear modulus of diabetic RBCs to be 5 times larger than the normal RBCs (*μ*_*d*_ = 5*μ*_0_).

**Fig 6:**
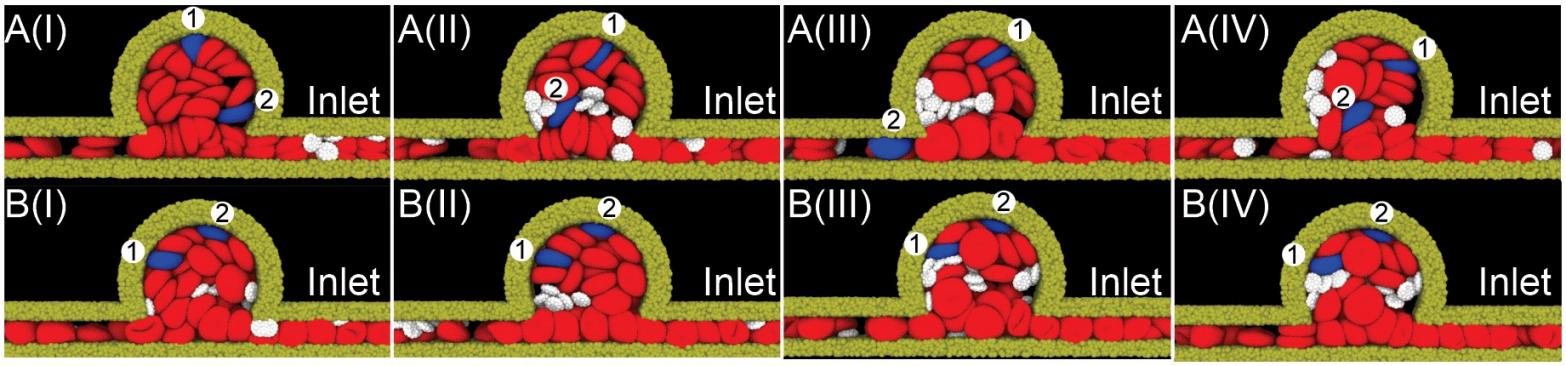
Sequential snapshots of RBCs (red) and platelets (white) transiting in the microchannel with MA2 (BNR = 5.4) under an inlet blood flow velocity of 1.5 mm/s. The timestamp increases sequentially from snapshot (I) to (IV). Two RBCs are highlighted in blue to track their motion during the simulation time. The results in (A) show notable motion of two tracer RBCs in the case of no RBC-RBC adhesion whereas no apparent motion is observed when RBC-RBC adhesion is considered in (B). Solvent particles are omitted for clarity.

**Fig 7:**
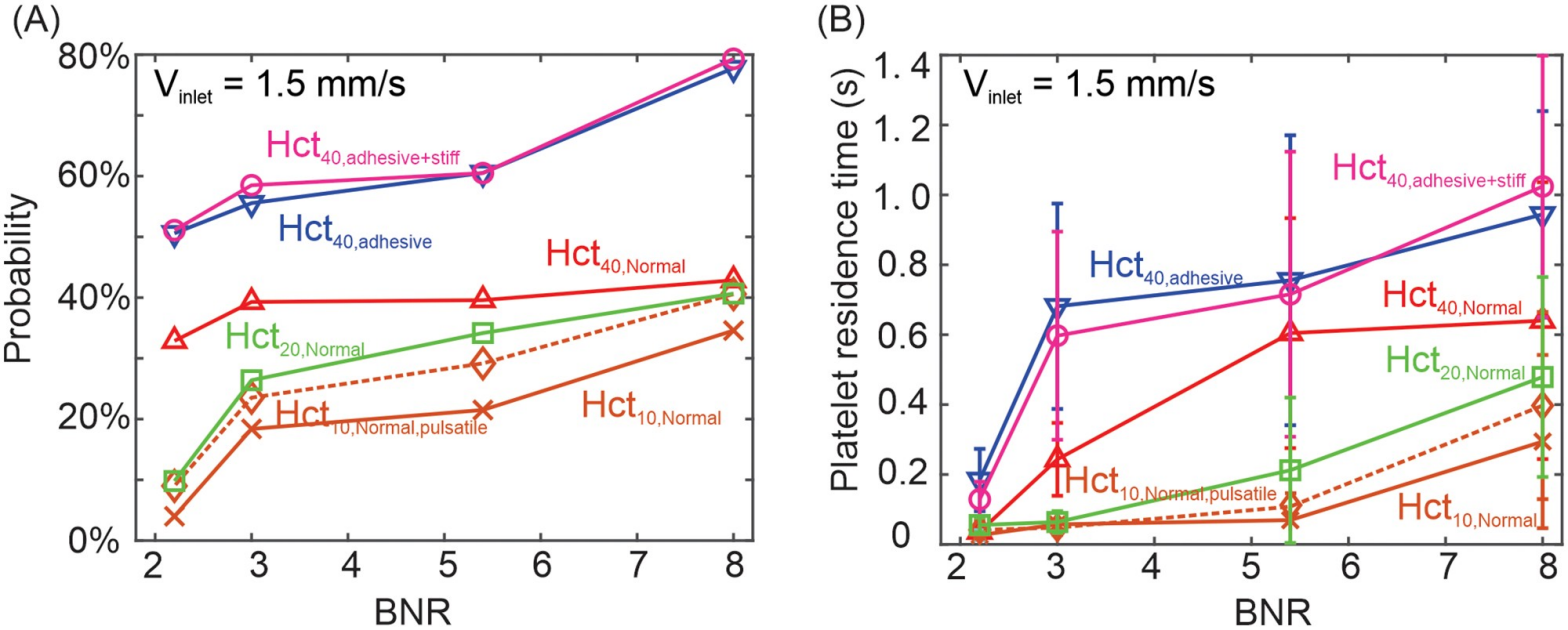
Effects of RBC-RBC adhesion, RBC stiffness, hematocrit level and pulsatile flow pattern on the transport of platelets in the MA channels. (A) Probability of platelets entering the MA lumen at an inlet blood flow velocity of 1.5 mm/s. Six different scenarios are examined, namely (i) RBCs without adhesion (red curve), (ii) RBCs with adhesion (blue curve), (iii) RBC with adhesion and increased stiffness (pink curve), (iv) normal RBCs with hematocrit level of 20% (green curve), (v) normal RBCs with hematocrit level of 10% (brown curve), (vi) normal RBCs with hematocrit level of 10% under pulsatile flow (brown dotted curve). (B) Residence time of platelets within the MAs at an inlet blood flow velocity of 1.5 mm/s. The same six scenarios as (A) are examined. The error bars are computed based on measurements of 20 platelets.

First, we explore the effect of RBC adhesion on the platelet transport in the four MA channels by adding adhesive forces between the RBCs with normal stiffness. The inlet blood flow velocity is selected to be 1.5 mm/s. Our simulation results show that RBC adhesion can slow down the motion of RBCs in the lumen of larger MAs (BNR = 8 and 5.4). As illustrated in Fig 6A(I)–6A(IV), there are notable motion of two highlighted RBCs (in blue) inside the MAs when no RBC adhesion is implemented. However, once the RBC-RBC adhesion is employed, Fig 6B(I)–6B(IV) show that the motion of RBCs inside the MA becomes negligible. Furthermore, Fig 7A (blue curve) shows that RBC-RBC adhesion increases the likelihood of platelets entering the MAs as it promotes the formation of RBC aggregates which could impose a stronger expulsion to the platelets and force them move towards the MA lumen. This effect is also observed in normal vessels where enhanced platelet margination is induced in blood flow with increased RBC aggregation [94–96]. Next, we increase the stiffness of the RBCs while maintaining the same level of RBC adhesion. Our results in Fig 7A (pink curve) show that the role of increased stiffness of RBCs in platelets transport in the MA channel is marginal. This finding is consistent with a prior study showing that the influence of RBC deformability on the collisions between RBCs and platelets was negligible [97]. We also compute the residence time of the platelets within MAs when the RBCs adhesion and increased RBC stiffness are considered. Fig 7B shows that RBC-RBC adhesion also can prolong the residence time of platelets in all four examined MAs whereas an increase in the stiffness of the adhesive RBCs does not further extend the residence of platelets.

### Effects of microhematocrit and pulsatility of blood flow on platelet transport

Prior studies have shown that hematocrit and blood flow rates in the retinal capillaries are greater than those in other capillary beds [98], which is probably caused by a higher level of oxygen consumption of the inner retina [99]. A recent *in vivo* experimental study [100] reported that the local hematocrit levels in the retinal microvasculature could vary significantly between 43% to 24% likely due to plasma skimming occurring at the bifurcations [101–105]. To investigate the impact of the variation of hematocrit levels on the blood cell transport, we reduce the hematocrit level in the microchannels from 40% to 20% and 10%, and compute the resulting probability of platelets entering the four examined MAs as well as their residence time inside at a blood velocity of 1.5 mm/s in the parent vessels. In addition, we examine how the pulsatility of the blood flow in the parent vessels affects the dynamics of blood cells inside MAs.

**Fig 8:**
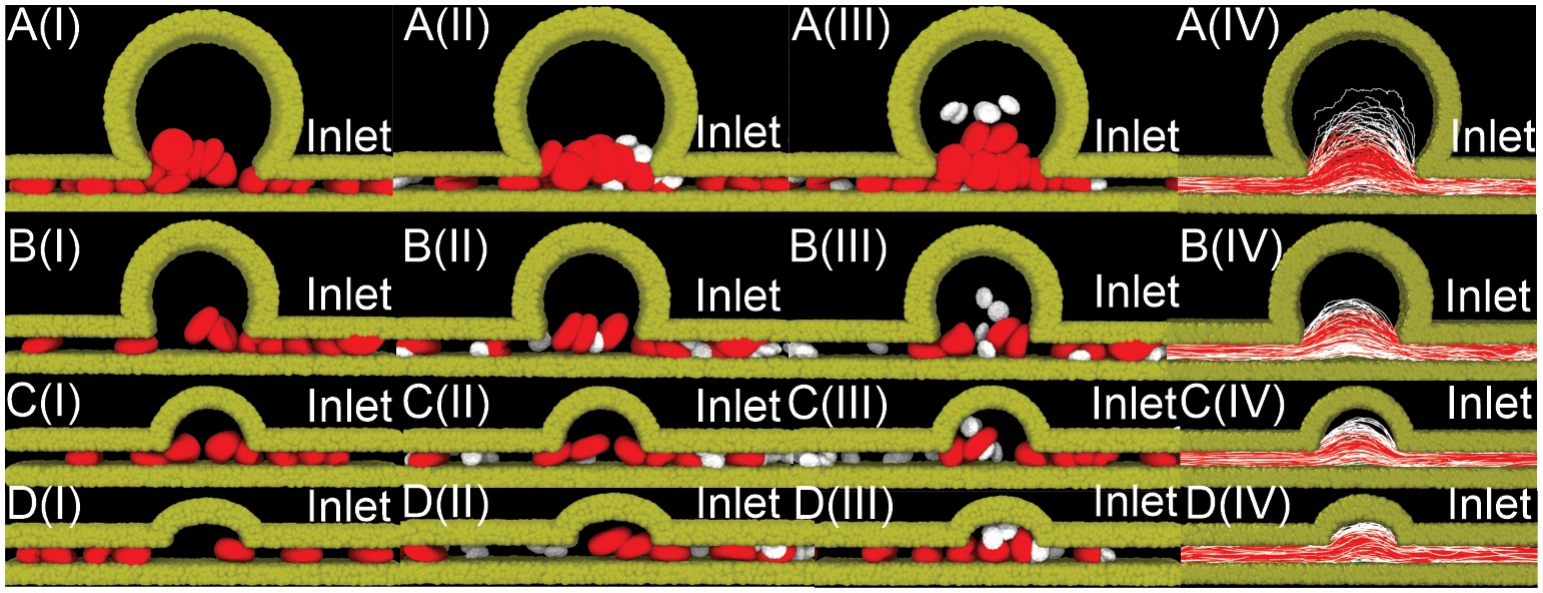
Sequential snapshots of RBCs (red) and platelets (white) traveling in the four MA microchannels with BNR = (A) 8, (B) 5.4, (C) 3, (D) 2.2, during the simulation time of 1.3 seconds. The hematocrit level is 10% in the microchannel. The timestamp increases sequentially from snapshot (I) to (III). RBCs are initially placed in the parent vessels. Solvent particles are omitted for clarity. The corresponding trajectories of RBCs (red curves) and platelets (white curves) are plotted in (IV). The inlet (right end of the microchannel) blood flow velocity is 1.5 mm/s for all the four MAs.

Sequential snapshots of RBCs and platelets transiting in the four MA microchannels with a hematocrit level of 10% are illustrated in Fig 8. These results show that RBCs, which are initially placed in the parent vessels of the MAs, flow in and exit the MAs without penetrating into the MA body for the MA channels with BNR = 5.4 and 8, consistent with the findings for the cases when the MA body is filled with RBCs at a hematocrit of 40% (see Fig 3). In contrast, platelets travel into the MA body occasionally, but at a lower frequency than the simulations with a hematocrit of 40% for the four examined MAs due to the reduced interaction with RBCs (see brown curve vs red curve in Fig 7A). As shown in Fig 7B, the residence times of the platelets inside the MAs with BNR = 3, 5.4 and 8 are also decreased when the hematocrit level is reduced to 10% and 20%, respectively. The trajectories of the RBCs in Fig 8A–8D(IV) further show that the main blood flow from the parent vessel of the MAs can only perfuse the MA with the smallest lumen (see Fig 8D), but cannot do so for the rest three MAs with larger BNR (see Fig 8A–8C). Next, we change the blood flow velocity in the parent vessels from a static to a pulsatile pattern with a mean velocity of 1.5 *mm*/*s*, following the measurement in [106]. The level of hematocrit is selected to be 10%. As shown by the brown curves in Fig 7A and 7B, the pulsatile flow pattern elevates the probability of platelets entering the four examined MAs as well as extends their residence time inside the MAs compared to the static flow pattern. However, its impact is less pronounced than the case where the level of hematocrit is increased from 10% to 20% (see green curves in Fig 7A and 7B).

## Discussion and summary

MAs are hallmark lesions in DR and a better understanding of their pathophysiological course might improve our ability to predict anatomic and visual outcomes in the diabetic eyes. Although recent advances in AOSLO imaging enable classification of the MA’s morphologies [6], detection of MA intraluminal thrombus [7] and assessment of the blood velocity fluctuations in the surrounding capillaries [53], the pathophysiology of hemodynamics in diabetic retinal MAs is still not fully understood. In this work, we model the RBCs and platelets flowing in retinal microvasculature containing MAs through particle-based simulations, aiming to investigate how the size of MAs, blood flow velocity, hematocrit levels and biomechanics of RBCs affect the transport of RBCs and platelets within MAs. Our results show that under the same inlet blood velocity, platelets are not only prone to enter MAs with larger BNR, but also undergo prolonged residence time in those MAs, both of which could contribute to platelet activation, adhesion and eventual thrombus formation inside MAs. Our results further indicate that increased inlet blood velocities and hematocrit level can boost the likelihood of platelets entering MAs as well as increase their residence time. Moreover, our simulations suggest that the enhanced RBC-RBC adhesion, such as in diabetic blood [77, 87], also promotes the migration of platelets into the MAs and prolongs platelet residence time. These simulation results demonstrate the essential roles of MA size, blood flow velocity and biomechanics of RBCs in regulating the transport of platelets in microvessels containing MAs.

The present simulation results also provide new insights and quantitative details to rationalize a variety of clinical findings pertaining to MAs in DR. For example, our results illustrate that for MAs with larger BNR (BNR = 8 and 5.4 in Fig 3A and 3B), there exist regions inside the MAs that are not perfused by the main blood flow from the parent channel inlet. As a result, endothelial cells within the MA body may become hypoxic, particularly when the hematocrit levels in the parent vessels are low, thereby exacerbating cellular inflammation and dysfunction and potentially leading to the leakage or rupture of the MAs. This result is consistent with a prior study showing that the BNR value is positively correlated with the level of dysfunction of endothelium, which is manifested by the increased vWF expression [9]. The hypoxic condition is likely to become more pronounced in diabetic blood as enhanced cell adhesion promotes RBC aggregation and thus enlarges the cell-free layer near the vessel walls [107–109], causing a reduced average hematocrit level in the branching vessels. Thus, the average hematocrit of the blood in capillaries is lower in diabetic patients than that of normal subjects, offering a mechanistic explanation for prior findings showing that RBC aggregation and rouleaux formation can cause hypoxia within microvasculature in diabetes [110–112].

We also note that the sluggish motion of blood cells in the nonperfused region could facilitate the thrombus formation in the MAs with large BNR. This result provides a rationale for the observations that blood clotting occurs more frequently in saccular-shaped MAs whose BNR can be as high as ∼15 [7]. Presence of a non-perfused region within MAs may also prevent timely delivery of platelets and necessary clotting factors to the injured endothelial cells and it is therefore possible that the thrombus or blood cell aggregates formed inside an MA may not have the same physiological function as in the hemostasis within normal vasculature. This explains why there is no clear correlation between the formation of thrombus and the risk of the MA leakage or rupture. Similarly, the heterogeneous role of thrombosis has been reported in various types of macro-scale aneurysms, such as cerebral aneurysms [32], abdominal aortic aneurysms [34, 113] and dissecting aortic aneurysms [42], where they can either degrade the vessel wall and thus accelerate aneurysm rupture or stabilize the aneurysm by reducing the wall shear stress. In case of two MAs with smaller BNR (BNR = 3 and 2.2), the lumen of MAs are fully perfused such that the RBCs and platelets can theoretically be transported to the potential injured endothelial cells inside MAs and form thrombus. This process might be associated with the disappearance of MAs in the retinas of diabetic patients over time [5], which may result from the full thrombosis of the dilated MA lumen.

There are several limitations in our current study. First, we simulate the blood flow in idealized MA geometries so that we can dissect and quantify the impacts of hemodynamics, MA geometries, hematocrit levels as well as RBC biomechanics on the blood cell transport in the MAs. Future simulations using realistic 3D geometries of MAs reconstructed from the AOSLO images will be performed to provide more physiological interpretation of thrombosis in MAs. In addition, we did not consider platelet adhesion and aggregation inside the MAs as the underlying mechanism is still not well-understood. The hypothesis that the hypoxic conditions in the MA could contribute to the leaking or rupture of MAs is derived based on the observations from our simulations and requires further experimental validation. We hope that these simulation-based hypotheses can potentially stimulate and steer new *in vitro* and *in vivo* studies in this area.

Altogether, we employ particle-based models, which explicitly represent the RBCs and platelets as well as their interaction, to simulate the RBC and platelet transport in the microchannels containing MAs, a process that is difficult to observe *in vivo* and hard to simulate using continuum-based computational models. We quantify the impacts of the size of MAs, blood flow velocity, hematocrit levels and biomechanics of RBCs on the probability of platelet entering MAs as well as their residence time inside the MAs and our simulation results provide new insights into a variety of clinical findings pertaining to MAs in DR. The current study suggests that computational simulations using particle-based modeling can be employed to improve our understanding of the pathogenesis of thrombosis inside MAs in DR.

## Supporting information

S1 Text Hydrodynamics and blood cell models. Table A. DPD fluid parameters used in the simulations. Table B. Cell membrane parameters for normal RBCs, diabetic RBCs and platelets. Table C. Parameters for interaction between different types of DPD particles. Table D. Morse potential parameters for cell-cell interactions.
(PDF)
