# Supplementary material for: Computational investigation of blood cell transport in retinal microaneurysms
Source: PLoS Comput Biol. 2022 Jan 5;18(1):e1009728. doi: 10.1371/journal.pcbi.1009728 (PMC8730408; doi:10.1371/journal.pcbi.1009728)
Supplement: S1 Text — Table A. DPD fluid parameters used in the simulations. Table B. Cell membrane parameters for normal RBCs, diabetic RBCs and platelets. Table C. Parameters for interaction between different types of DPD particles. Table D. Morse potential parameters for cell-cell interactions. (PDF) [file pcbi.1009728.s001.pdf]

Computational investigation of blood cell transport in retinal microaneurysms

He Li, Yixiang Deng, Konstantina Sampani, Shengze Cai, Zhen Li, Jennifer K. Sun, George E. Karniadakis.

Supporting Information (SI)

S1 Text. Hydrodynamics and blood cell models

We employ dissipative particle dynamics (DPD) method to model blood flow, *i.e.*, plasma, red blood cells (RBCs) and platelets, in the microfluidic channels. The DPD method is a mesoscopic particle-based simulation technique, where each DPD particle represents a lump of molecules and interacts with other particles through soft pairwise forces that depend only on their relative positions and velocities [1, 2]. Therefore, DPD can provide the correct hydrodynamic behavior of fluids at the mesoscale, and it has been successfully applied to study complex fluids [3, 4]. The equation of motion for each particle i is governed by the sum of pair interactions $\mathbf{f}_i$ with the surrounding particles j and integrated using a velocity-Verlet algorithm. The time evolution of velocity ($\mathbf{v}_i$) and position ($\mathbf{r}_i$) of a particle i with mass m_i is determined by Newton's second law of motion:

$$\mathrm{d}\mathbf{r}_i = \mathbf{v}_i\,\mathrm{dt}\,; \quad \mathrm{d}\mathbf{v}_i = \mathbf{f}_i/m_i\,\mathrm{dt}. \tag{1}$$

In the classical DPD method, the total force $\mathbf{f}_i$ exerted on particle i by particle j is composed of a conservative force ($\mathbf{F}_{ij}^{C}$), a dissipative force ($\mathbf{F}_{ij}^{D}$), and a random force ($\mathbf{F}_{ij}^{R}$) given by

$$\mathbf{F}_{ij}^{C} = a_{ij}(1-\frac{r_{ij}}{r_c})\hat{\mathbf{r}}_{ij} \quad \text{for} \quad r_{ij} \leq r_c; \quad \mathbf{F}_{ij}^{C} = 0 \quad \text{for} \quad r_{ij} > r_c, \tag{2}$$

$$\mathbf{F}_{ij}^{D} = \gamma\omega_d(r_{ij})(\hat{\mathbf{r}}_{ij}\cdot\hat{\mathbf{v}}_{ij})\hat{\mathbf{r}}_{ij}, \tag{3}$$

$$\mathbf{F}_{ij}^{R} = \sigma\omega_r(r_{ij})\frac{\zeta_{ij}}{\sqrt{dt}}\hat{\mathbf{r}}_{ij}, \tag{4}$$

where r_c is a cut-off radius, and a_{ij}, γ, σ are the conservative, dissipative, random coefficients, respectively, r_{ij} is the distance with the corresponding unit vector $\hat{\mathbf{r}}_{ij}$, $\hat{\mathbf{v}}_{ij}$ is the difference between the two velocities, ζ_{ij} is a Gaussian random number with zero mean and unit variance, and dt is the simulation timestep size. The parameters γ and σ and the weight functions coupled through the fluctuation-dissipation theorem and are related by $\omega_d = \omega_r^2$ and $\sigma^2 = 2\gamma k_B T$, where k_B is the Boltzmann constant and T is the temperature of the system. The weight function $\omega_r(r_{ij}) = (1 - r_{ij}/r_c)^k$ with $k = 1$ in the standard DPD method, whereas other values of k have been used to increase the fluid viscosity [5]. Table A presents the DPD parameters used for the fluid particles in the current work. More detailed description of DPD method can be found in [2, 6].

Table A. DPD fluid parameters used in current study: n is the fluid's number density, a_c is the conservative force coefficient, γ is the dissipative force coefficient and k is the weight function exponent. In all simulations, we set the particle mass $m = 1$, and the thermal energy $k_B T = 0.1$ in DPD units.

n	r_c	a_C	γ	k
4	2.0	5.0	30.0	0.2

In addition to blood plasma modeled by collections of free DPD particles, the membrane of suspending cells including RBCs and platelets is constructed by a 2D triangulated network with N_v vertices (DPD particles). The vertices are connected by N_s elastic bonds to impose proper membrane mechanics. These DPD representations of RBCs and platelets were extensively used and validated in the previous studies for both healthy and diseased cells [7–11]. For a single cell, the free energy (V_{cell}) is given by

$$V_{cell} = V_s + V_b + V_{a+v}. \tag{5}$$

The elastic energy V_s representing the elastic interactions of the cell membrane is defined by

$$V_s = \sum_{j \in 1 \ldots N_s} \left[\frac{k_{\mathrm{B}} T l_m (3x_j^2 - 2x_j^3)}{4p(1 - x_j)} + \frac{k_p}{l_j} \right], \tag{6}$$

where p is the persistence length, k_p is the spring constant, $k_{\mathrm{B}}T$ is the energy unit, l_j is the length of the spring j, l_m is the maximum spring extension, and $x_j = l_j / l_m$. p and k_p are computed by balancing the forces at equilibrium and from their relation to the macroscopic shear modulus, μ_s:

$$\mu_s = \frac{\sqrt{3} k_{\mathrm{B}} T}{4 p l_m x_0} \left(\frac{x_0}{2(1 - x_0)^3} - \frac{1}{4(1 - x_0)^2} + \frac{1}{4} \right) + \frac{3\sqrt{3} k_p}{4 l_0^3}, \tag{7}$$

where l_0 is the equilibrium spring length and $x_0 = l_0 / l_m$. The bending resistance V_b of the cell membrane is modeled by

$$V_b = \sum_{j \in 1 \ldots N_s} k_b \left[1 - \cos(\theta_j - \theta_0) \right], \tag{8}$$

where k_b is the bending constant, and it is related to the macroscopic bending rigidity k_c with the expression $k_b = 2k_c/\sqrt{3}$, θ_j is the instantaneous angle between two adjacent triangles having the common edge j, and θ_0 is the spontaneous angle. In addition, the area and volume constraints V_{a+v} are imposed to mimic the area-preserving lipid bilayer and the incompressible interior fluid. The corresponding energy is given by

$$V_{a+v} = \sum_{j \in 1 \ldots N_t} \frac{k_d (A_j - A_0)^2}{2A_0} + \frac{k_a (A_{\mathrm{cell}} - A_0^{\mathrm{tot}})^2}{2A_0^{\mathrm{tot}}} + \frac{k_v (V_{\mathrm{cell}} - V_0^{\mathrm{tot}})^2}{2V_0^{\mathrm{tot}}}, \tag{9}$$

where N_t is the number of triangles in the membrane network, A_0 is the equilibrium value of a triangle area, and k_d, k_a and k_v are the local area, global area and volume constraint coefficients, respectively. The terms A_0^{tot} and V_0^{tot} are targeted cell area and volume.
In this study, we model a normal RBC with N_v = 500, shear modulus μ_0 = 4.73 μN/m and bending rigidity k_0 = 2.4×10^{-19} J. The cell surface area is selected to be A_0^{tot} = 132.9 μm^2, and cell volume V_0^{tot} = 92.5 μm^3, which give surface to volume ratio S/V = 1.44. All parameters

Table B. Cell membrane parameters for normal RBCs (NRBCs), RBCs with increased stiffness (DRBCs) and platelets with mean platelet volume (MPV)= 6 fL (PLTs). N_v is the number of DPD particles on the membrane, l_m is the maximum bond extension, l_0 is the equilibrium bond length, k_b is the bending constant, μ_s is the shear modulus, A_0^{tot} and V_0^{tot} are the specified cell area and volume, respectively, $k_d + k_a$ is the combined area constraint coefficient, and k_v is the volume constraint coefficient.

cell	N_v	l_m/l_0	k_b	μ_s	A_0^{tot} (V_0^{tot})	$k_d + k_a$ (k_v)
NRBC	500	1.8	6.025	100.0	132.87 (92.45)	5000 (5000)
DRBC	500	1.8	6.025	500.0	132.87 (92.45)	5000 (5000)
PLT	48	1.8	600.0	10^4	19.63 (6.02)	5000 (10^4)

used in our RBC model are listed in Table B and they are calibrated based on existing experimental data and validated with simulations, from single RBC mechanics to blood flow dynamics [3, 7, 12, 13].
In the case of platelets, which are nearly rigid in their passive form, we choose shear modulus and bending rigidity sufficiently large (100 times larger than the normal RBCs) to ensure its rigid behavior. The number of vertices in the platelet's membrane network is N_v = 48 and the aspect ratio of the cell is AR = 0.38. Based on our previous analysis on the patient-specific data [12], a normal platelet has cell volume V_0^{tot} = 6 μm^3. The DPD parameters used in eqn (2)-(4) for all types of DPD particles and the cell membrane parameters used in eqn (6)-(9) for all blood cell models are given in Tables B and C, respectively.

Table C. Parameters for interaction between different types of DPD particles. r_c is a cut-off radius, a_{ij} is the conservative coefficient, γ is the dissipative coefficient, and k is the weight function exponent. In all simulations, we set the particle mass $m = 1$, and the thermal energy $k_BT = 0.10$ in DPD units. Note that S stands for solvent (representing plasma), R stands for NRBCs and DRBCs whereas P stands for platelets.

type	r_c	a_{ij}	γ	k
S-S	2.0	5.0	30.0	0.20
S-R	1.5	0.0	45.0	0.20
S-P	1.5	0.0	10.0	0.20
R-R	1.0	10.0	10.0	0.20
R-P	1.0	10.0	10.0	0.20
P-P	1.0	10.0	10.0	0.20

In order to relate the DPD parameters with the physical values, we need to first define length and time scales. The RBC membrane shear modulus imposes the time scale for the DPD system, which follows

$$[t] = [L]\frac{\eta^P}{\eta^M}\frac{\mu_s^M}{\mu_s^P} \tag{10}$$

where μ_s is the RBC membrane shear modulus, η is the plasma viscosity, and superscripts M

and P denote the model (DPD) and physical units, respectively. The length scale is taken as $[L] = 1 \times 10^{-6}m$, whereas the time scale is evaluated to be $[t] = 2.6 \times 10^{-5}s$ (using membrane shear modulus of healthy RBCs $\mu_s^p = 4.73 \times 10^{-6}N/m$ and plasma viscosity $\eta^P = 1.2 \times 10^{-3}Pa \cdot s$).
In order to prevent cell overlap we also employ a Morse potential between cell membrane particles in the form of

$$V_M(r) = D_e[e^{2\beta(r_0-r)} - 2e^{\beta(r_0-r)}]\,, \tag{11}$$

where r is the separation distance, r_0 is the zero force distance, D_e is the well depth of the potential, and β characterizes the interaction range. By properly setting the parameters, we can ensure sufficiently strong repulsive forces between cell membrane particles and prevent their overlap. We present the Morse potential parameters used for cell-cell interactions in Table D. Note that the cutoff radius $r_{cut} = 1$ is given for all the Morse interactions when RBC-RBC adhesion is not considered. When RBC-RBC adhesion is considered, r_{cut} for RBC-RBC interaction is selected to be 1.2, following the work of Deng et al. [13], while maintaining $r_{cut} = 1$ for other pairs of interactions.

Table D. Morse potential parameters for cell-cell interactions. D_e is the well depth of the potential, r_0 is the zero force distance, and β characterizes the interaction range. Note that R stands for NRBCs and DRBCs whereas P stands for platelets.

type	D_e	β	r_0
R-R	10.0	1.5	1.0
R-P	10.0	1.5	1.0
P-P	10.0	1.5	1.0

References

1. Espanol P, Warren P. Statistical mechanics of dissipative particle dynamics. EPL (Europhysics Letters). 1995;30(4):191.
2. Espanol P, Warren P. Statistical mechanics of dissipative particle dynamics. Europhys Lett. 1995;30(4):191.
3. Fedosov DA, Pan W, Caswell B, Gompper G, Karniadakis GE. Predicting human blood viscosity in silico. Proc Natl Acad Sci USA. 2011;108(29):11772–11777.
4. Ye T, Phan-Thien N, Lim CT. Particle-based simulations of red blood cells —- a review. J Biomech. 2016;49(11):2255–2266.
5. Fan X, Phan-Thien N, Chen S, Wu X, Yong Ng T. Simulating flow of DNA suspension using dissipative particle dynamics. Phys Fluids. 2006;18(6):063102.
6. Groot RD, Warren PB. Dissipative particle dynamics: bridging the gap between atomistic and mesoscopic simulation. J Chem Phys. 1997;107(11):4423–4435.
7. Fedosov DA, Caswell B, Karniadakis GE. A multiscale red blood cell model with accurate mechanics, rheology, and dynamics. Biophys J. 2010;98(10):2215–2225.
8. Pivkin IV, Karniadakis GE. Accurate coarse-grained modeling of red blood cells. Phys Rev Lett. 2008;101(11):118105.

9. Yazdani A, Karniadakis GE. Sub-cellular modeling of platelet transport in blood flow through microchannels with constriction. Soft Matter. 2016;12(19):4339–4351.

10. Lei H, Karniadakis GE. Probing vasoocclusion phenomena in sickle cell anemia via mesoscopic simulations. Proc Natl Acad Sci USA. 2013;110(28):11326–11330.

11. Cai S, Li H, Zheng F, Kong F, Dao M, Karniadakis GE, et al. Artificial intelligence velocimetry and microaneurysm-on-a-chip for three-dimensional analysis of blood flow in physiology and disease. Proceedings of the National Academy of Sciences of the United States of America. 2021;10(1):1–11.

12. Chang H, Li X, Karniadakis GE. Modeling of biomechanics and biorheology of red blood cells in type 2 diabetes mellitus. Biophysical Journal. 2017;113(2):481–490.

13. Deng Y, Papageorgiou DP, Li X, Perakakis N, Mantzoros CS, Dao M, et al. Quantifying Fibrinogen-Dependent Aggregation of Red Blood Cells in Type 2 Diabetes Mellitus. Biophys J. 2020;119(5):900–912.
